# Dysregulation of type 2 innate lymphoid cells and T_H_2 cells impairs pollutant-induced allergic airway responses

**DOI:** 10.1016/j.jaci.2016.03.044

**Published:** 2017-01

**Authors:** Katrien C. De Grove, Sharen Provoost, Rudi W. Hendriks, Andrew N.J. McKenzie, Leen J.M. Seys, Smitha Kumar, Tania Maes, Guy G. Brusselle, Guy F. Joos

**Affiliations:** a Department of Respiratory Medicine, Laboratory for Translational Research in Obstructive Pulmonary Diseases, Ghent University Hospital, Ghent, Belgium; b Department of Pulmonary Medicine, Erasmus MC, Rotterdam, The Netherlands; c MRC Laboratory of Molecular Biology, Francis Crick Avenue, Cambridge, United Kingdom

**Keywords:** Diesel exhaust particles, house dust mite, type 2 innate lymphoid cell, T_H_2 response, asthma, AHR, Airway hyperresponsiveness, BALF, Bronchoalveolar lavage fluid, DC, Dendritic cell, DEPs, Diesel exhaust particles, HDM, House dust mite, ILC2, Type 2 innate lymphoid cell, MHCII, MHC class II, MLN, Mediastinal lymph node, Rag, Recombination-activating gene, RORα, RAR-related orphan receptor α, TCR, T-cell receptor, TSLP, Thymic stromal lymphopoietin, WT, Wild type

## Abstract

**Background:**

Although the prominent role of T_H_2 cells in type 2 immune responses is well established, the newly identified type 2 innate lymphoid cells (ILC2s) can also contribute to orchestration of allergic responses. Several experimental and epidemiologic studies have provided evidence that allergen-induced airway responses can be further enhanced on exposure to environmental pollutants, such as diesel exhaust particles (DEPs). However, the components and pathways responsible remain incompletely known.

**Objective:**

We sought to investigate the relative contribution of ILC2 and adaptive T_H_2 cell responses in a murine model of DEP-enhanced allergic airway inflammation.

**Methods:**

Wild-type, Gata-3^+/nlslacZ^ (Gata-3–haploinsufficient), RAR-related orphan receptor α (RORα)^fl/fl^IL7R^Cre^ (ILC2-deficient), and recombination-activating gene (Rag) 2^−/−^ mice were challenged with saline, DEPs, or house dust mite (HDM) or DEP+HDM. Airway hyperresponsiveness, as well as inflammation, and intracellular cytokine expression in ILC2s and T_H_2 cells in the bronchoalveolar lavage fluid and lung tissue were assessed.

**Results:**

Concomitant DEP+HDM exposure significantly enhanced allergic airway inflammation, as characterized by increased airway eosinophilia, goblet cell metaplasia, accumulation of ILC2s and T_H_2 cells, type 2 cytokine production, and airway hyperresponsiveness compared with sole DEPs or HDM. Reduced Gata-3 expression decreased the number of functional ILC2s and T_H_2 cells in DEP+HDM-exposed mice, resulting in an impaired DEP-enhanced allergic airway inflammation. Interestingly, although the DEP-enhanced allergic inflammation was marginally reduced in ILC2-deficient mice that received combined DEP+HDM, it was abolished in DEP+HDM-exposed Rag2^−/−^ mice.

**Conclusion:**

These data indicate that dysregulation of ILC2s and T_H_2 cells attenuates DEP-enhanced allergic airway inflammation. In addition, a crucial role for the adaptive immune system was shown on concomitant DEP+HDM exposure.

## Graphical abstract

**Figure:**
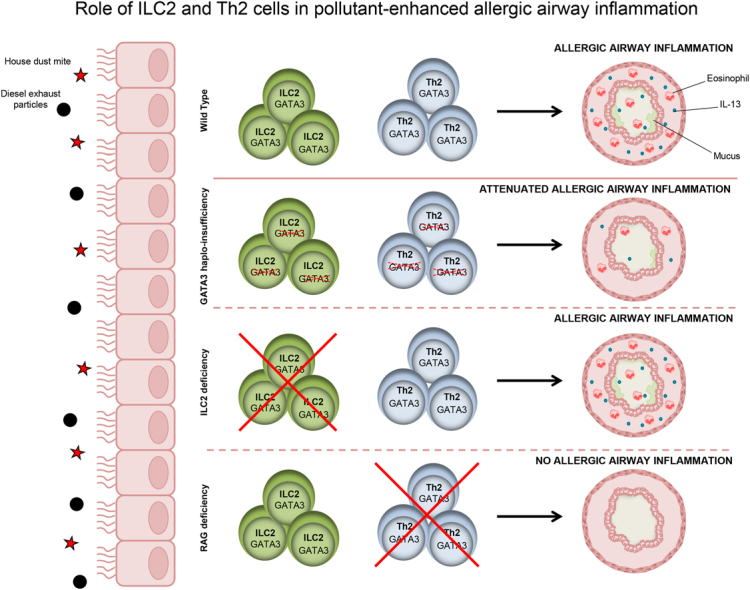

Asthma is a chronic disorder of the conducting airways associated with reversible airway obstruction, chronic airway inflammation, airway remodeling, and airway hyperresponsiveness (AHR).^1^ It is a heterogeneous disease in which multiple phenotypes can be distinguished based on clinical characteristics and the inflammatory profile. Asthma that originates during childhood (early-onset asthma) mostly has an atopic component^2^, ^3^ and is typically considered a T_H_2-driven disease.^4^

In addition to the adaptive immune system, the airway epithelium has gained great importance during initiation and maintenance of the allergic and asthmatic cascade. In particular, it has been shown that on allergen exposure, several epithelial cytokines, such as IL-25, IL-33, and thymic stromal lymphopoietin (TSLP), are involved in the pathogenesis of asthma.^5^, ^6^ Moreover, several genes discovered in genome-wide association studies (ie, IL-33, IL-1RL1, and TSLP) support a key role for these cytokines.^7^, ^8^ On the one hand, these epithelium-derived cytokines have the capability to activate the adaptive immune system by stimulating T_H_2-polarizing dendritic cells (DC).^5^ On the other hand, the recently identified type 2 innate lymphoid cells (ILC2s) also become activated by these cytokines.^9^, ^10^, ^11^ Analogous with T_H_2 cells, ILC2s require the transcription factor Gata-3 and are a potent source of the type 2 cytokines IL-5 and IL-13, which are able to induce lung eosinophilia and mucus hypersecretion.^11^, ^12^, ^13^, ^14^, ^15^ Studies in recombination-activating gene (Rag)^−/−^ mice have shown that these ILC2s are crucial players in allergic airway responses.^16^ Even in the absence of the adaptive immune system, ILC2s were able to mediate eosinophilia, goblet cell metaplasia, type 2 cytokine production, and AHR.^17^, ^18^, ^19^ In addition, mice that were ILC2 deficient because of targeting of the transcription factor RAR-related orphan receptor α (RORα) had decreased type 2 immune responses.^14^, ^20^, ^21^ Interestingly, it was reported that ILC2s and T cells interact with each other and that this crosstalk could contribute to the maintenance, proliferation, and activation of both ILC2s and T_H_2 cells.^21^, ^22^, ^23^

In addition to allergen exposure, it has become well accepted that traffic-related particulate matter, such as diesel exhaust particles (DEPs), also contributes to the development and exacerbation of asthma.^24^, ^25^, ^26^ For instance, epidemiologic studies reported a correlation between high DEP levels and the frequency of symptomatic episodes in allergic children.^27^ In addition, combined allergen plus DEP administration during controlled human exposure studies resulted in increased allergen-specific immunoglobulin levels and type 2 cytokine responses.^28^ Furthermore, concomitant DEP plus house dust mite (HDM) exposure in murine models enhanced eosinophilia, immunoglobulin production, AHR, and remodeling.^29^ However, the mechanisms underlying the enhanced effects of DEPs on allergen-induced airway inflammation remain largely unknown. Several studies suggested that the airway epithelium could be an important player because particulate matter was also able to stimulate the release of several epithelium-derived cytokines, such as TSLP and IL-33, which can lead to enhanced DC maturation and T_H_2 responses.^30^, ^31^, ^32^, ^33^ However, whether this also activates ILC2s is unknown.

In this article we investigate the relative contribution of ILC2s and the adaptive immune system in the enhancing effects of DEPs on allergen-induced airway inflammation. We show in a murine model that concomitant exposure to a clinically relevant allergen (ie, HDM) and DEPs enhances several allergic airway responses, including airway eosinophilia, goblet cell metaplasia, increased ILC2 and T_H_2 cell numbers, type 2 cytokine production, and AHR. Because Gata-3 is an important transcription factor during the development and function of both ILC2s and T_H_2 cells,^34^ we used haploinsufficient Gata-3^+/nlslacZ^ mice, which have a reduced expression of Gata-3,^35^ to demonstrate that the enhancing effects of DEPs on allergic airway inflammation depend on Gata-3–mediated regulation of ILC2s and T_H_2 cells. Moreover, to examine the specific contribution of ILC2s in the model of DEP-enhanced allergic airway inflammation, we used a conditionally targeted RORα^fl/fl^ mouse that, when intercrossed with IL-7 receptor–Cre mice, yields an ILC2-deficient mouse strain in which other lineages are unaffected.^21^ Finally, to investigate the functional role of the adaptive immune system in this model, we used Rag2^−/−^ mice, which lack mature T and B cells.^36^ We demonstrated that ILC2s marginally contributed to DEP-enhanced allergic airway responses, whereas the adaptive immune system appeared critical to orchestrate the enhanced effect of DEPs on allergic airway inflammation and AHR.

## Methods

### Mice

Female C57BL/6 mice (6-8 weeks old) were obtained from the Jackson Laboratory (Bar Harbor, Me). C57BL/6 Gata-3^+/nlslacZ^ mice and their wild-type (WT) littermates were a kind gift of Dr R. Hendriks (Department of Pulmonary Medicine, Erasmus MC, Rotterdam, The Netherlands)^35^ and bred in our animal facility at Ghent University. RORα^fl/fl^IL7R^Cre^ mice and RORα^fl/fl^IL7R^+/+^ control mice were on a C57BL/6 background.^21^ C57BL/6 Rag2^−/−^ mice^36^ and their WT control mice were purchased from Taconic (Hudson, NY). All *in vivo* manipulations were approved by the Animal Ethical Committee of the Faculty of Medicine and Health Sciences of Ghent University.

### Intranasal instillation of reagents

DEPs (SRM 2975) were purchased from the National Institute for Standards and Technology. HDM *(Dermatophagoides pteronyssinus)* was obtained from Greer Laboratories (Lenoir, NC). Saline, 1 μg of HDM extract dissolved in saline, 25 μg of DEPs suspended in saline, or a combination of DEP+HDM was delivered intranasally to isoflurane-anesthetized mice by using a continuous flow vaporizer on days 1, 8, and 15. Two days after the last challenge, mice were killed with a lethal dose of intraperitoneal pentobarbital.

### Bronchoalveolar lavage fluid

A tracheal cannula was inserted, and bronchoalveolar lavage fluid (BALF) was recovered by means of instillation of 3× 300 μL of 1% HBSS supplemented with 1% BSA and 6× 500 μL of HBSS supplemented with EDTA. The lavage fractions were pooled, and total cell counts were measured with a Bürker chamber. Differential cell counts were performed on cytospin preparations after May-Grünwald-Giemsa staining. The remaining cells were used for flow cytometry.

### Lung and mediastinal lymph node single-cell suspensions

The pulmonary circulation was rinsed with saline supplemented with EDTA to remove the intravascular pool of cells. Lungs and mediastinal lymph nodes (MLNs) were minced and incubated for 45 minutes in digestion medium (RPMI-1640 supplemented with 5% FCS, 2 mmol/L l-glutamine, 0.05 mmol/L 2-mercaptomethanol, 100 U/mL penicillin, 100 μg/mL streptomycin, 1 mg/mL collagenase type 2, and 0.02 mg/mL DNase I) at 37°C and 5% CO_2_. Red blood cells were lysed with ammonium chloride buffer. Total cell counts were performed with a Z2 Coulter Counter (Beckman Coulter, Fullerton, Calif).

### MLN cell culture

MLNs were harvested and digested, as described above. Cells were cultured in culture medium either alone or supplemented with 3.75 μg/well HDM in round-bottom 96-well plates and incubated in a humidified 37°C incubator in a 5% CO_2_ atmosphere. After 5 days, supernatants was harvested for cytokine measurements.

### Flow cytometry

**Fig E1:**
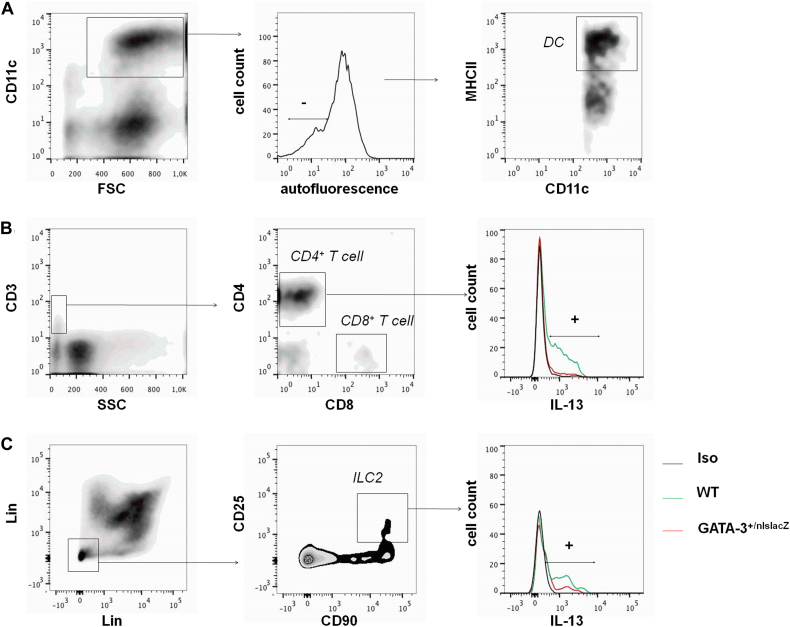
Representative density plots and gating strategy of all analyzed cell populations in BALF. WT mice were exposed to 25 μg of DEPs plus 1 μg of HDM. **A,** DCs were gated as CD11c^high^, low autofluorescent, and MHCII^+^. **B,** CD4^+^ T cells were CD3^+^, CD4^+^, and CD8^−^. CD8^+^ T cells were characterized as CD3^+^, CD4^−^, and CD8^+^. Intracellular IL-13 production of CD4^+^ T cells was investigated. **C,** ILC2s were identified as Lin^−^ (CD3^−^, CD5^−^, NK1.1^−^, TCRβ^−^, CD11c^−^, CD11b^−^, and CD45R^−^), CD25^+^, and CD90^+^ cells. Intracellular IL-13 production of ILC2s was investigated. *FSC*, Forward scatter; *SSC*, side scatter.

**Fig E2:**
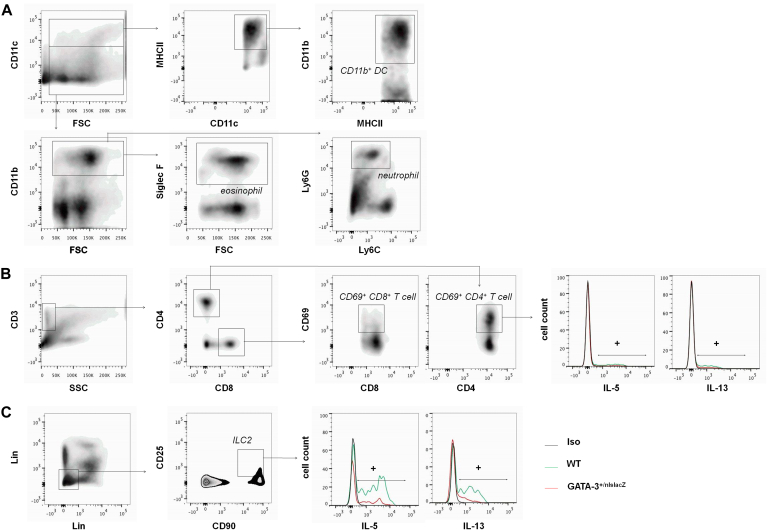
Representative density plots and gating strategy of all analyzed cell populations in lung tissue. WT mice were exposed to 25 μg of DEPs plus 1 μg of HDM. **A,** Pulmonary CD11b^+^ DCs were identified as CD11c^+^, MHCII^+^, and CD11b^+^. Eosinophils were gated as CD11c^−^, CD11b^+^, and Siglec-F^+^. Neutrophils were CD11c^−^, CD11b^+^, Ly6G^+^, and Ly6C^+^. **B,** CD4^+^ T cells were gated as CD3^+^, CD4^+^, CD8^−^, and CD69^+^. CD8^+^ T cells were characterized as CD3^+^, CD4^−^, CD8^+^, and CD69^+^. Intracellular IL-5 and IL-13 production of CD4^+^ T cells was investigated. **C,** ILC2s were identified as Lin^−^ (CD3^−^, CD5^−^, NK1.1^−^, TCRβ^−^, CD11c^−^, CD11b^−^, and CD45R^−^), CD25^+^, and CD90^+^. Intracellular IL-5 and IL-13 production of ILC2s was investigated. *FSC*, Forward scatter; *SSC*, side scatter.

BALF cells and single lung suspensions were stained with a combination of anti-mouse fluorochrome-conjugated mAbs against CD4 (GK1.5), CD8 (53-6.7), CD11b (M1/70), CD69 (H1.2F3), Ly6C (AL-21), Ly6G (1A8), MHC class II (MHCII; 2G9), Siglec-F (E50-2440; all from BD Biosciences, San Jose, Calif); CD3 (145-2C11), CD90.2 (30.H12; all from BioLegend, San Diego, Calif); and CD5 (53-7.3), CD11c (N418), CD25 (PC61.5), CD127 (A7R34), CD45R (RA3-6B2), NK1.1 (PK136), and T-cell receptor (TCR) β (H57-597; all from eBioscience, San Diego, Calif). For cytoplasmic cytokine staining, cells were stimulated for 4 hours with ionomycin and phorbol 12-myristate 13-acetate supplemented with brefeldin A and monensin at 37°C for 4 hours. The intracellular fixation and permeabilization buffer set (eBioscience) was used for fixation and cell permeabilization. The following antibodies were used: phycoerythrin-conjugated anti–IL-5 (TRFK5), anti–IL-13 (eBio13A), and isotype-matched controls (eBioscience). Data acquisition was performed on a FACSCalibur flow cytometer running CellQuest software or an LSR II cytometer running DIVA software. Two hundred fifty thousand events were collected. Cell subsets were analyzed with FlowJo software (TreeStar, Ashland, Ore). Representative flow cytometric density plots and the gating strategy of all analyzed cell populations in BALF and lung tissue are shown in Figs E1 and E2 in this article's Online Repository at www.jacionline.org, respectively.

### Histology

The left lung was fixated with 4% paraformaldehyde and embedded in paraffin. Three-micrometer transverse sections were stained with Congo Red to identify eosinophils or with periodic acid–Schiff to analyze goblet cells. Quantitative measurements were performed with a Zeiss KS400 Image analyzer platform (Zeiss, Oberkochen, Germany).

### Protein measurements

IL-4, IL-5, and IL-13 levels in BALF or MLN supernatants were measured by using commercially available ELISA kits (R&D Systems, Minneapolis, Minn). In lung homogenates IL-25 and IL-33 levels were measured with ELISA (R&D Systems), according to the manufacturer's instructions. HDM-specific IgG_1_ levels were determined on serum collected by means of retro-orbital bleeding. For detection, plates were coated with HDM extract. Serum was added, followed by a horseradish peroxidase–conjugated polyclonal goat anti-mouse IgG_1_ antibody (Bethyl Laboratories, Montgomery, Tex). The plate was read at 490 nm. HDM-IgG_1_ levels were reported in OD. All samples were on the same plate, and experimental data from different plates are not presented together.

### AHR

AHR in response to increasing doses of carbachol (0, 20, 40, 80, 160, 320, and 640 μg/kg) was measured 48 hours after the last intranasal instillation by using the forced oscillation technique (flexiVent System; SCIREQ, Montreal, Quebec, Canada). Neuromuscular blockade was induced by injecting 1 mg/kg pancuronium bromide intravenously. A “snapshot perturbation” maneuver was imposed to measure the resistance (R) of the whole respiratory system (airways, lung, and chest wall).

### Statistical analysis

Statistical analysis was performed with SPSS software (version 22.0; SPSS, Chicago, Ill). Nonparametric tests (Kruskal-Wallis and Mann-Whitney *U* tests) were used to compare different groups, according to standard statistical criteria. Values were reported as means ± SEMs. *P* values of less than .05 were considered significant.

## Results

### Exposure to DEPs enhances HDM-induced airway inflammation

**Fig E3:**
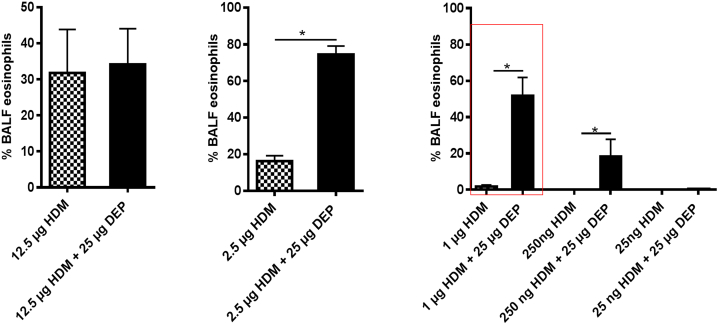
Titration of the HDM dose for the combined DEP+HDM model. C57BL/6 mice were exposed to decreasing doses of only HDM (ie, 12.5 μg, 2.5 μg, 1 μg, 250 ng, and 25 ng of HDM) or the combination 25 μg of DEPs plus decreasing doses of HDM on days 1, 8, and 15. On day 17, BALF eosinophils were determined on cytospin preparations. Data presented on the different graphs represent separate experiments. The *red box* represents the selected dose for the experiments in the article. Results are expressed as means ± SEMs (n = 8 mice per group). *P < .05.

**Fig E4:**
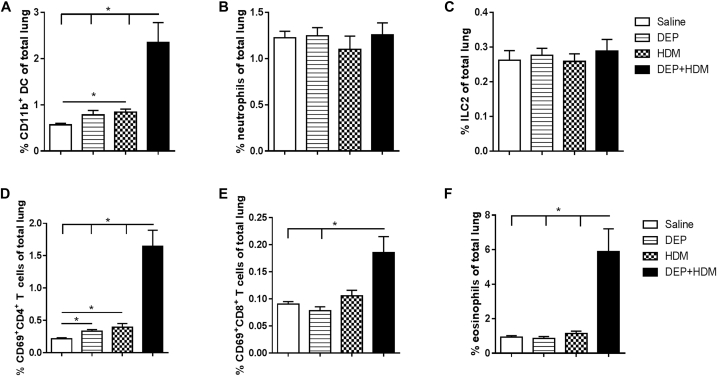
Adjuvant effect of DEPs on HDM-induced airway inflammation in the lung. WT mice were exposed to saline *(white bars)*, 25 μg of DEPs *(striped bars)*, 1 μg of HDM *(checked bars)*, or DEP+HDM *(black bars)* on days 1, 8, and 15. **A-F,** DCs (CD11c^+^MHCII^+^CD11b^+^; Fig E4, *A*), neutrophils (CD11c^−^CD11b^+^Ly6G^+^Ly6C^+^; Fig E4, *B*), eosinophils (CD11c^−^CD11b^+^Siglec-F^+^; Fig E4, *C*), ILC2s (Lin^−^ [CD3^−^, CD5^−^, NK1.1^−^, TCRβ^−^, CD11c^−^, CD11b^−^, and CD45R^−^] CD25^+^CD90^+^; Fig E4, *D*), CD4^+^ T cells (CD3^+^CD4^+^CD8^−^CD69^+^; Fig E4, *E*), and CD8^+^ T cells (CD3^+^CD8^+^CD4^−^CD69^+^; Fig E4, *F*) in lung tissue were determined by using flow cytometry. Results are expressed as the mean percentages of total lung ± SEM (n = 7-8 mice per group). *P < .05.

**Fig 1:**
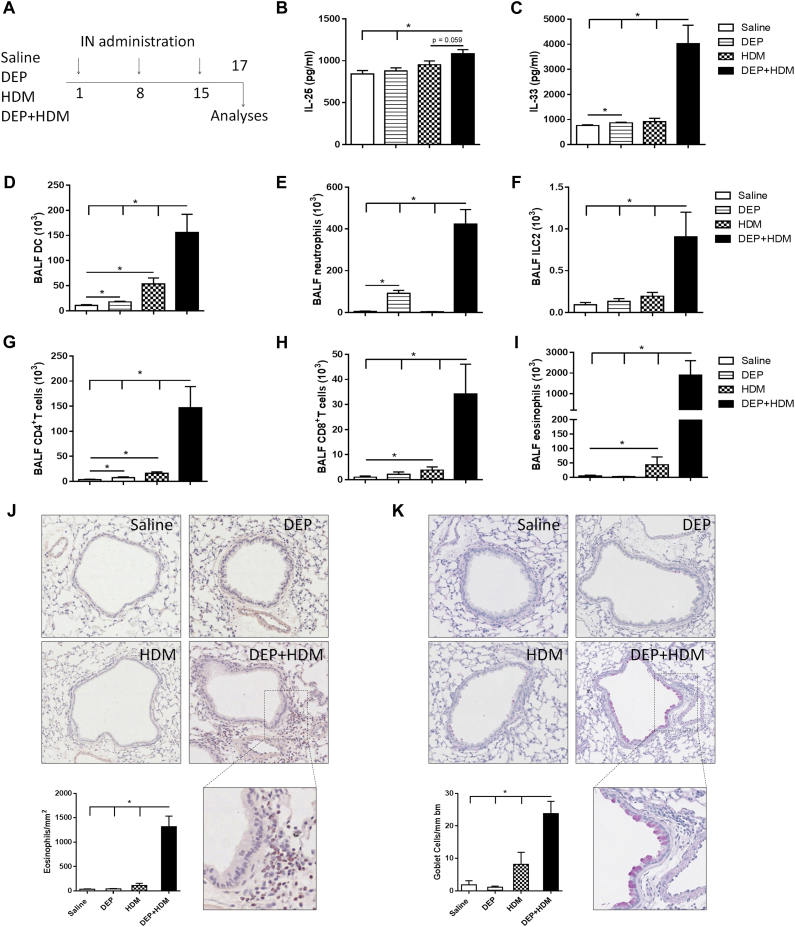
Exposure to DEPs enhances HDM-induced airway inflammation. WT mice were exposed to saline *(white bars)*, 25 μg of DEPs *(striped bars)*, 1 μg of HDM *(checked bars)*, or DEP+HDM *(black bars)* on days 1, 8, and 15. **A,** Schematic overview of our model of DEP-enhanced HDM-induced airway inflammation. *IN*, Intranasal. **B** and **C,** IL-25 (Fig 1, *B*) and IL-33 (Fig 1, *C*) protein levels in lungs were determined by using ELISA. **D-I,** DCs (CD11c^high^, low autofluorescent, and MHCII^+^; Fig 1, *D*), neutrophils (Fig 1, *E*), ILC2s (Lin^−^ [CD3^−^, CD5^−^, NK1.1^−^, TCRβ^−^, CD11c^−^, CD11b^−^, and CD45R^−^] CD25^+^CD90^+^; Fig 1, *F*), CD4^+^ T cells (CD3^+^CD4^+^CD8^−^; Fig 1, *G*), CD8^+^ T cells (CD3^+^CD8^+^CD4^−^; Fig 1, *H*), and eosinophils (Fig 1, *I*) in BALF were determined by using flow cytometry, except neutrophils and eosinophils, which were determined on cytospin preparations. **J** and **K,** Representative photomicrographs and quantification of peribronchovascular eosinophils (Fig 1, *J*) and periodic acid–Schiff–stained lung specimens (Fig 1, *K*). Results are expressed as means ± SEMs (n = 7-8 mice per group). *P < .05. Data are representative of 3 independent experiments. Representative flow cytometric density plots and gating strategy are shown in Fig E1.

C57BL/6 mice were exposed to saline, DEPs alone, HDM alone or combined DEP+HDM (Fig 1, *A*). Mice were exposed to doses of DEPs and HDM that elicited almost no inflammatory response on their own to have a model in which we could optimally examine the potential adjuvant capacities of DEPs on HDM-induced airway inflammation (dose titrations for HDM are shown in Fig E3 in this article's Online Repository at www.jacionline.org, BALF inflammation to low vs high doses of DEPs was previously shown^32^). Exposure to DEPs alone slightly increased numbers of DCs, neutrophils, and CD4^+^ T cells in the BALF in comparison with those in saline-exposed mice (Fig 1, *D*, *E*, and *G*). Administration of HDM induced a modest increase in DC, CD4^+^ T-cell, CD8^+^ T-cell, and eosinophil numbers in BALF when compared with those in the saline group (Fig 1, *D* and G-I). In contrast, combined exposure to DEP+HDM greatly enhanced the allergic airway immune responses. Concomitant exposure to DEP+HDM elicited a marked increase in levels of the epithelium-derived cytokines IL-25 and IL-33 (Fig 1, *B* and *C*). Moreover, numbers of DCs, neutrophils, ILC2s, CD4^+^ T cells, CD8^+^ T cells, and eosinophils were significantly increased in the BALF of WT mice that received combined DEP+HDM compared with those in the 3 control groups (Fig 1, *D-I*). Of note, all ILC2s expressed ST2 (data not shown), resembling natural ILC2s.^37^ Coexposure of DEP+HDM was also associated with a significant enhancement of inflammatory cells in the lung tissue (see Fig E4 in this article's Online Repository at www.jacionline.org). Furthermore, histologic examination revealed increased peribronchial eosinophilic inflammation and goblet cell metaplasia on simultaneous DEP+HDM exposure (Fig 1, *J* and *K*).

### Combined exposure to DEP+HDM increases type 2 cytokine production and HDM-specific IgG_1_ levels and induces AHR

**Fig 2:**
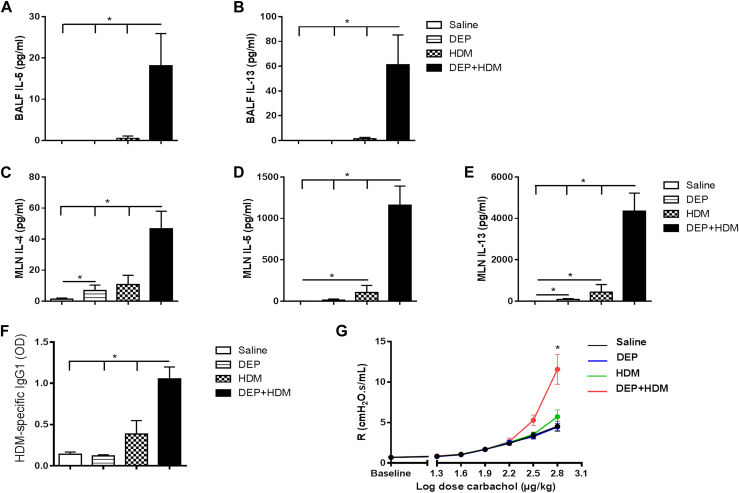
Combined exposure to DEP+HDM increases type 2 cytokine production and HDM-specific IgG_1_ levels and induces AHR. WT mice were exposed to saline *(white bars)*, 25 μg of DEPs *(striped bars)*, 1 μg of HDM *(checked bars)*, or DEP+HDM *(black bars)* on days 1, 8, and 15. **A** and **B,** IL-5 (Fig 2, *A*) and IL-13 (Fig 2, *B*) protein levels in BALF were determined by using ELISA. **C-E,** IL-4 (Fig 2, *C*), IL-5 (Fig 2, *D*), and IL-13 (Fig 2, *E*) protein levels in the supernatants of HDM-restimulated MLNs were determined by using ELISA. **F,** HDM-specific IgG_1_ titers in serum were determined by using ELISA. **G,** Airway resistance *(R)* of mice exposed to saline *(black line)*, DEPs *(blue line)*, HDM *(green line)*, and DEP+HDM *(red line)* was measured in response to increasing doses of carbachol. Results are expressed as means ± SEMs (n = 7-8 mice per group). *P < .05. Data are representative of 2 independent experiments.

Typical type 2 cytokines were evaluated in the BALF and supernatants of HDM-restimulated MLNs obtained from WT mice exposed to saline, DEPs, HDM or combined DEP+HDM. In the BALF of mice exposed to DEP+HDM, increased IL-5 and IL-13 levels were found compared with the 3 control groups, whereas HDM or DEPs alone elicited no response (Fig 2, *A* and *B*). In the supernatants of HDM-restimulated MLNs, concomitant DEP+HDM-exposed mice had markedly higher IL-4, IL-5, and IL-13 levels compared with those in the control groups (Fig 2, *C-E*). In contrast, exposure to DEPs alone was associated with a modest increase in IL-4 and IL-13 levels in comparison with those in the saline group (Fig 2, *C* and *E*). Sole HDM administration slightly increased IL-5 and IL-13 levels in the MLNs compared with those in the saline-exposed group (Fig 2, *D* and *E*). Furthermore, combined DEP+HDM-exposed mice had significantly increased HDM-specific IgG_1_ titers in serum when compared with those in saline-, DEPs-, and HDM-exposed control groups (Fig 2, *F*). Additionally, mice that were concomitantly exposed to DEP+HDM showed AHR in comparison with the 3 control groups (Fig 2, *G*).

### Reduced Gata-3 expression impairs airway eosinophilia and mucus metaplasia on combined DEP+HDM exposure

**Fig 3:**
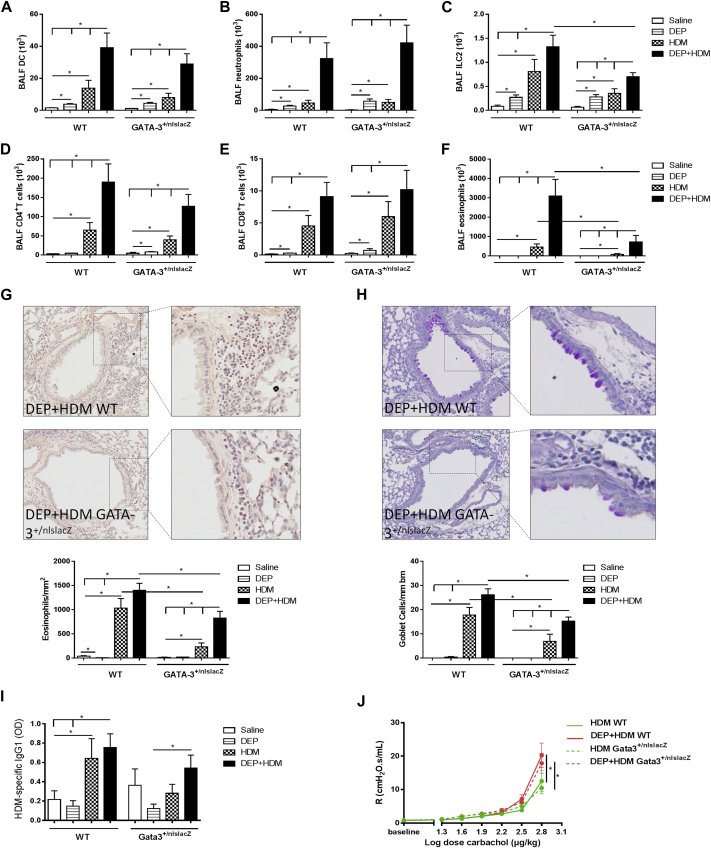
Reduced Gata-3 expression impairs airway eosinophilia and mucus metaplasia on combined DEP+HDM exposure. WT and Gata-3^+/nlslacZ^ mice were exposed to saline *(white bars)*, 25 μg of DEPs *(striped bars)*, 1 μg of HDM *(checked bars)*, or DEP+HDM *(black bars)*. **A-F,** DCs (CD11c^high^, low autofluorescent, and MHCII^+^; Fig 3, *A*), neutrophils (Fig 3, *B*), ILC2s (Lin^−^ [CD3^−^, CD5^−^, NK1.1^−^, TCRβ^−^, CD11c^−^, CD11b^−^, and CD45R^−^] CD25^+^CD90^+^; Fig 3, *C*), CD4^+^ T cells (CD3^+^CD4^+^CD8^−^; Fig 3, *D*), CD8^+^ T cells (CD3^+^CD8^+^CD4^−^; Fig 3, *E*), and eosinophils (Fig 3, *F*) in BALF were determined by using flow cytometry, except neutrophils and eosinophils, which were determined on cytospin preparations. **G** and **H,** Representative photomicrographs and quantification of Congo Red–stained lungs (Fig 3, *G*) or periodic acid–Schiff–stained mucus-producing goblet cells (Fig 3, *H*) of DEP+HDM-exposed WT and Gata-3^+/nlslacZ^ mice. **I,** HDM-specific IgG_1_ levels in serum were determined by using ELISA. **J,** Airway resistance *(R)* in WT *(full line)* and Gata-3^+/nlslacZ^*(broken line)* mice was measured in response to increasing doses of carbachol. Results are expressed as means ± SEMs (n = 7-9 mice per group). *P < .05.

Gata-3 is an important transcription factor for the development of ILC2s and T_H_2 cells.^34^ Because the Gata-3 gene copy number is positively correlated with both ILC2 and T_H_2 function,^38^, ^39^, ^40^ we evaluated the effect of reduced Gata-3 expression in our model of DEP-enhanced allergic airway inflammation. Gata-3^+/nlslacZ^ mice, in which 1 allele is disrupted by insertion of a β-galactosidase reporter,^35^ and WT littermates were exposed to saline, DEPs, HDM or the combination of DEP+HDM. Concomitant DEP+HDM exposure in Gata-3^+/nlslacZ^ mice resulted in DC, neutrophil, CD4^+^ T-cell, and CD8^+^ T-cell numbers that were comparable with those in WT littermates, whereas ILC2 and eosinophil numbers in the BALF were significantly reduced (Fig 3, *A-F*). Moreover, histologic analyses in DEP+HDM-exposed Gata-3^+/nlslacZ^ mice revealed a diminished peribronchial eosinophilic inflammation and goblet cell metaplasia compared with that seen in their littermate control mice (Fig 3, *G* and *H*). Also, on sole HDM exposure, reduced eosinophilia (Fig 3, *G*) and goblet cell metaplasia (Fig 3, *H*) were observed in the Gata-3^+/nlslacZ^ mice in comparison with their littermates. Comparable HDM-specific IgG_1_ levels in the serum were found in WT and Gata-3^+/nlslacZ^ mice that were exposed to DEP+HDM (Fig 3, *I*). Moreover, concomitant exposure to DEP+HDM led to a similar increase in airway responsiveness in Gata-3^+/nlslacZ^ mice and WT littermates when compared with that seen in their HDM control groups (Fig 3, *J*).

### Reduced Gata-3 expression decreases type 2 cytokine production by ILC2s and CD4^+^ T cells on combined DEP+HDM exposure

**Fig 4:**
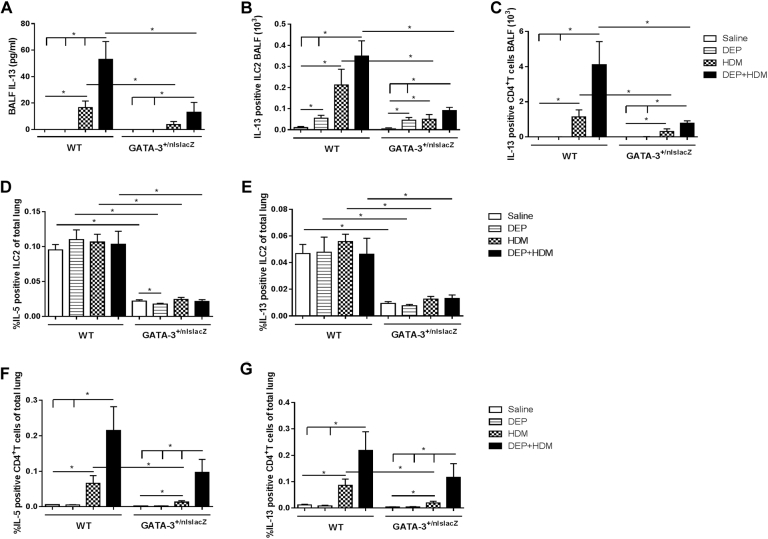
Reduced Gata-3 expression decreases cytokine production by ILC2s and CD4^+^ T cells on combined DEP+HDM exposure. WT and Gata-3^+/nlslacZ^ mice were exposed to saline *(white bars)*, 25 μg of DEPs *(striped bars)*, 1 μg of HDM *(checked bars)*, or DEP+HDM *(black bars)*. **A,** IL-13 protein levels in BALF were determined by using ELISA. **B-G,** BALF or lung cells were stimulated for 4 hours with phorbol 12-myristate 13-acetate/ionomycin, intracellularly labeled for cytokine production, and analyzed by using flow cytometry. Percentage of IL-13–expressing ILC2s (Lin^−^ [CD3^−^, CD5^−^, NK1.1^−^, TCRβ^−^, CD11c^−^, CD11b^−^, and CD45R^−^] CD25^+^CD90^+^; Fig 4, *B*) or CD4^+^ T cells (CD3^+^CD4^+^; Fig 4, *C*) in BALF; proportion of IL-5–producing (Fig 4, *D*) and IL-13–producing (Fig 4, *E*) ILC2s (Lin^−^ [CD3^−^, CD5^−^, NK1.1^−^, TCRβ^−^, CD11c^−^, CD11b^−^, and CD45R^−^] CD25^+^CD90^+^) in total lung tissue; and percentage of IL-5^+^ (Fig 4, *F*) and IL-13^+^ (Fig 4, *G*) T cells (CD3^+^CD4^+^) of total lungs are shown. Results are expressed as means ± SEMs (n = 7-8 mice per group). *P < .05. Representative flow cytometric histograms and density plots are shown in Figs E1 (BALF) and E2 (lung).

We further assessed the type 2 cytokine production in Gata-3^+/nlslacZ^ mice and WT littermates in response to combined DEP+HDM. Exposure to DEP+HDM led to reduced IL-13 levels in BALF of Gata-3^+/nlslacZ^ mice compared with levels seen in their littermates (Fig 4, *A*). In addition, intracellular type 2 cytokine production in both ILC2s and CD4^+^ T cells was investigated in BALF and lung tissue. The increased numbers of IL-13^+^ ILC2s and CD4^+^ T cells observed in BALF of DEP+HDM-exposed WT mice were significantly decreased in DEP+HDM-exposed Gata-3^+/nlslacZ^ mice (Fig 4, *B* and *C*). Moreover, IL-5– and IL-13–expressing ILC2s were significantly diminished in lung single-cell suspensions of Gata-3^+/nlslacZ^ mice independent of exposure compared with littermates (Fig 4, *D* and *E*). The increased percentages of IL-5^+^ and IL-13^+^ CD4^+^ T cells observed in the coexposed DEP+HDM Gata-3^+/nlslacZ^ mice did not significantly differ from those in their WT control mice. In contrast, Gata-3^+/nlslacZ^ mice exposed to HDM only had significantly lower IL-5^+^ and IL-13^+^ CD4^+^ T-cell numbers in comparison with those in their littermates (Fig 4, *F* and *G*).

### ILC2s marginally contribute to DEP-enhanced allergic airway inflammation

**Fig 5:**
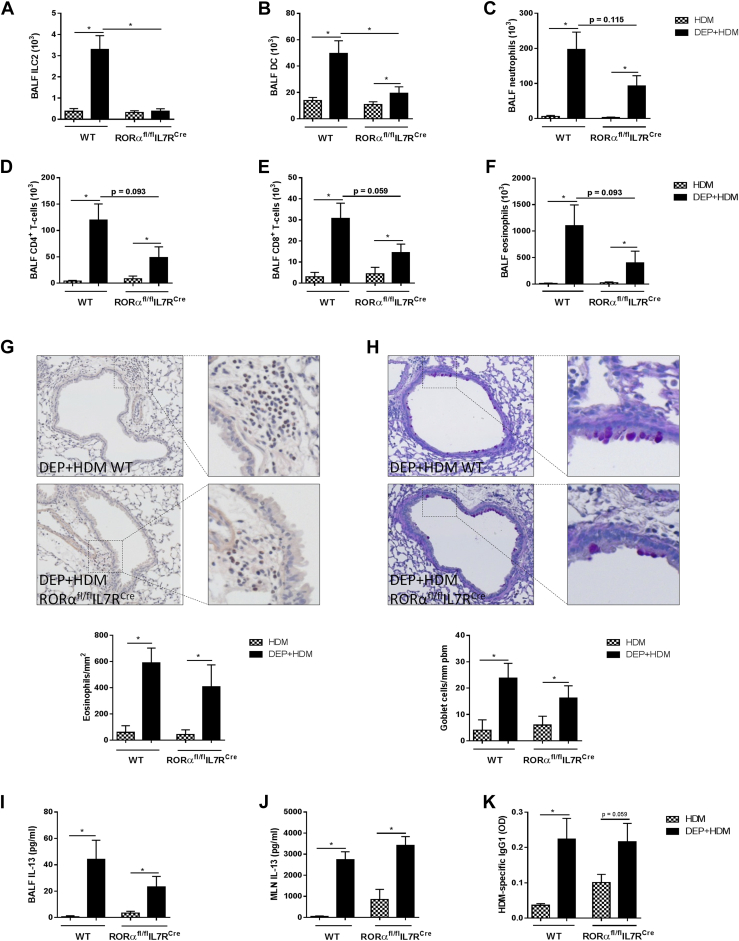
ILC2s marginally contribute to DEP-enhanced allergic airway inflammation. RORα^fl/fl^IL7R^Cre^ mice and RORα^fl/fl^IL7R^+/+^ WT control mice were exposed to 1 μg of HDM *(checked bars)* or DEP+HDM *(black bars)*. **A-F,** ILC2s (Lin^−^ [CD3^−^, CD5^−^, NK1.1^−^, TCRβ^−^, CD11c^−^, CD11b^−^, and CD45R^−^] CD25^+^CD90^+^; Fig 5, *A*), DCs (CD11c^high^, low autofluorescent, and MHCII^+^; Fig 5, *B*), neutrophils (Fig 5, *C*), CD4^+^ T cells (CD3^+^CD4^+^CD8^−^; Fig 5, *D*), CD8^+^ T cells (CD3^+^CD8^+^CD4^−^; Fig 5, *E*), and eosinophils (Fig 5, *F*) in BALF were determined by using flow cytometry, except neutrophils and eosinophils, which were determined on cytospin preparations. **G** and **H,** Representative photomicrographs and quantification of Congo Red–stained lungs (Fig 5, *G*) or periodic acid–Schiff–stained mucus-producing goblet cells (Fig 5, *H*). **I-K,** BALF IL-13 protein levels (Fig 5, *I*), IL-13 levels in the supernatants of HDM-restimulated MLNs (Fig 5, *J*), and HDM-specific IgG_1_ levels in serum (Fig 5, *K*) were determined by using ELISA. Results are expressed as means ± SEMs (n = 8 mice per group). *P < .05.

Because Gata-3 haploinsufficiency affects both the innate and adaptive components, we next examined the specific contribution of ILC2s in the enhancing effects of DEPs on allergic airway inflammation. For this, we exposed RORα^fl/fl^IL7R^Cre^ (ILC2-deficient) mice^21^ and their corresponding RORα^fl/fl^IL7R^+/+^ control mice to HDM and DEP+HDM. As expected, ILC2 numbers in BALF were abolished in RORα^fl/fl^IL7R^Cre^ mice independent of the exposure (Fig 5, *A*). Concomitant DEP+HDM-exposed RORα^fl/fl^IL7R^Cre^ mice had significantly reduced DCs in the BALF (Fig 5, *B*), whereas the numbers of BALF neutrophils, CD4^+^ T cells, CD8^+^ T cells, and eosinophils tended to decrease compared with those in RORα^fl/fl^IL7R^+/+^ control mice (Fig 5, *C-F*). Histologic analyses further revealed a similar peribronchial eosinophilia and goblet cell metaplasia between DEP+HDM-exposed RORα^fl/fl^IL7R^Cre^ mice and control mice (Fig 5, *G* and *H*). Moreover, the increased BALF IL-5 (data not shown) and IL-13 levels found on DEP+HDM exposure in the BALF and supernatants of restimulated MLNs did not differ between RORα^fl/fl^IL7R^Cre^ mice and RORα^fl/fl^IL7R^+/+^ control mice (Fig 5, *I* and *J*). HDM-specific IgG_1_ levels were also comparable between DEP+HDM-exposed RORα^fl/fl^IL7R^Cre^ mice and control mice (Fig 5, *K*).

### The adaptive immune system has a crucial role in DEP-enhanced allergic airway inflammation

**Fig 6:**
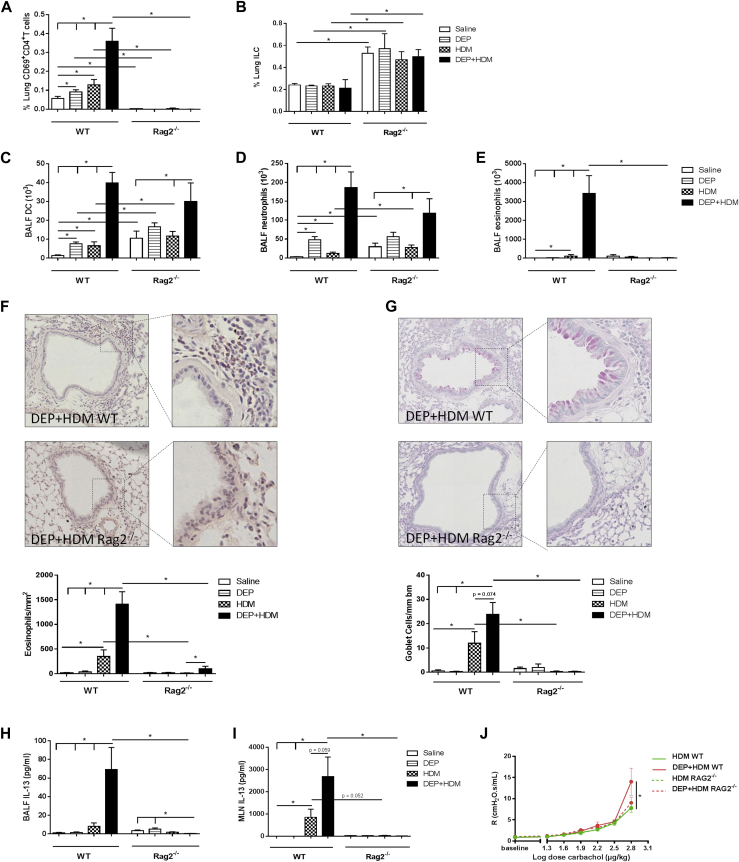
The adaptive immune system has a crucial role in DEP-enhanced allergic airway inflammation. WT and Rag2^−/−^ mice were exposed to saline *(white bars)*, 25 μg of DEPs *(striped bars)*, 1 μg of HDM *(checked bars)*, or DEP+HDM *(black bars)*. **A-E,** Percentage of CD4^+^ T cells (CD3^+^CD4^+^CD8^−^CD69^+^) of total lung (Fig 6, *A*), percentage of lung ILC2s (Lin^−^ [CD3^−^, CD5^−^, NK1.1^−^, TCRβ^−^, CD11c^−^, CD11b^−^, and CD45R^−^] CD25^+^CD127^+^; Fig 6, *B*), number of BALF DCs (CD11c^high^, low autofluorescent, and MHCII^+^; Fig 6, *C*), number of BALF neutrophils (Fig 6, *D*), and number of BALF eosinophils (Fig 6, *E*) were determined by using flow cytometry, except neutrophils and eosinophils, which were determined on cytospin preparations. **F** and **G,** Representative photomicrographs and quantification of Congo Red–stained lungs (Fig 6, *F*) or periodic acid–Schiff–stained mucus-producing goblet cells (Fig 6, *G*) of DEP+HDM-exposed WT and Rag2^−/−^ mice. **H** and **I,** BALF IL-13 protein levels (Fig 6, *H*) and IL-13 levels in the supernatants of HDM-restimulated MLNs (Fig 6, *I*) were determined by using ELISA. **J,** Airway resistance *(R)* in WT *(full line)* and Rag2^−/−^*(broken line)* mice was measured in response to increasing doses of carbachol. Results are expressed as means ± SEMs (n = 7-8 mice per group). *P < .05.

To investigate how the adaptive immune system contributes to the DEP-enhanced allergic airway inflammation, we exposed WT and Rag2^−/−^ mice, which lack an adaptive immune system,^36^ to saline, DEPs, HDM or combined DEP+HDM. As expected, Rag2^−/−^ mice had no mature CD4^+^ T cells in the lung, whereas the proportion of lung ILC2s was increased independent of the exposure (Fig 6, *A* and *B*). Rag2^−/−^ mice and WT control mice coexposed to DEP+HDM had similar ratios of DCs and neutrophils in the BALF (Fig 6, *C* and *D*). However, combined DEP+HDM exposure resulted in a complete abolishment of BALF eosinophils compared with the WT control mice (Fig 6, *E*). Histologic analyses further revealed a severely reduced eosinophilia and abolished goblet cell metaplasia in the Rag2^−/−^ mice that received combined DEP+HDM in comparison with values in WT control mice (Fig 6, *F* and *G*). Additionally, combined DEP+HDM exposure did not increase BALF IL-5 (data not shown) and IL-13 levels in BALF and supernatants of restimulated MLNs in Rag2^−/−^ mice compared with those in the WT control group (Fig 6, *H* and *I*). The modest inflammation that was seen in the WT mice on sole HDM exposure was also completely abolished in the Rag2^−/−^ mice. Independent of exposure, there was no HDM-specific IgG_1_ production in Rag2^−/−^ mice compared with that seen in WT control mice (data not shown). Furthermore, Rag2^−/−^ mice had no increase in airway responsiveness in response to DEP+HDM (Fig 6, *J*).

## Discussion

In this article we demonstrated that dysregulation in ILC2 and T_H_2 cell numbers and function by targeting Gata-3 was associated with an attenuated airway inflammation on concomitant DEP+HDM exposure. Moreover, we showed a critical role of the adaptive immune system to DEP-enhanced allergic responses and AHR, whereas ILC2s only marginally contribute to DEP-enhanced allergic airway inflammation.

The immunologic mechanisms by which DEPs can promote allergen-induced airway inflammation are largely unknown. To unravel these cellular and molecular mechanisms, we set up a mouse model with concomitant exposure to a clinically relevant allergen (ie, HDM) and DEPs. To have an optimal model in which the potential adjuvant effects of DEPs on HDM-induced allergic airway inflammation can be evaluated, we downtitrated the dose of DEPs and HDM until they elicited minimal inflammatory responses on their own. We demonstrated that concomitant exposure to DEP+HDM markedly enhanced multiple features of allergic inflammation, as characterized by an eosinophilic response, goblet cell metaplasia, ILC2 and T_H_2 cell accumulation, type 2 cytokine production, increased HDM-specific IgG_1_ levels, and AHR. In accordance with our findings, several experimental and epidemiologic studies already provided evidence regarding the synergistic ability of DEPs in allergic airway inflammation.^25^, ^27^, ^28^, ^29^ Although these studies already demonstrated the enhanced effects on airway remodeling, AHR, eosinophilic inflammation, and immunoglobulin production, insights concerning the number and function of the recently identified ILC2s in response to concomitant exposure to DEP+HDM were lacking.

Epithelial cells are the first barrier to encounter several inhaled allergens and particles. In response to these particulates, the epithelium can release cytokines and chemokines to direct the recruitment and activation of several innate and adaptive immune cells.^5^, ^6^ On HDM, for instance, it was shown that IL-25 and IL-33 levels were upregulated, contributing to the observed airway and lung inflammation.^41^, ^42^ Although we observed no increased IL-25 and IL-33 response on exposure to DEPs or HDM only, combined DEP+HDM exposure elicited an increase in IL-25 and IL-33 levels in particular, suggesting that DEPs work synergistically with HDM to induce release of epithelial cytokines in the environment.

These epithelium-derived cytokines share the capacity to stimulate T_H_2 development by polarizing DCs on the one hand^5^ and activating ILC2s on the other hand.^9^, ^10^ Although the role of T_H_2 cells in the pathogenesis of asthma is well established,^43^ it was recently shown that ILC2s can also contribute substantially to allergic airway inflammation.^16^ Moreover, ILC2s can mediate AHR independent of the adaptive immune system.^18^, ^19^ Importantly, we found that exposure to DEP+HDM increased the number of cytokine-expressing ILC2s and T_H_2 cells in the alveolar space, suggesting that type 2 cytokine production of both cell types contributes to the adjuvant effects of DEPs on allergic airway inflammation. Although the numbers of type 2–expressing ILC2s in the lung did not increase on combined DEP+HDM exposure, ILC2s could still be critical in initiating and maintaining DEP-enhanced type 2 immune responses. At least in response to the pollutant ozone, it was suggested that the increased activation of ILC2s was associated with an enhanced eosinophilic inflammation toward *Aspergillus fumigatus*.^44^

Because Gata-3 is an important transcription factor during the development and function of ILC2s, as well as T_H_2 cells,^34^ we investigated the effects of Gata-3 modulation in our model of DEP-enhanced allergic airway inflammation. At baseline, Gata-3^+/nlslacZ^ mice had fewer functional ILC2s in the lung, whereas effects on T_H_2 cells were limited. In response to DEP+HDM, both IL-13–expressing ILC2s and Th2 cells were attenuated in BALF of Gata-3^+/nlslacZ^ mice, suggesting that the observed reduction in type 2 airway inflammation could be the result of diminished functional ILC2 and T_H_2 cell numbers in the bronchoalveolar space. In addition, considering that coexposure of DEP+HDM only tended to decrease the type 2 cytokine–expressing T_H_2 cells in the lung, whereas ILC2s were abolished, the imbalance of functional ILC2s and T_H_2 cells in the lung could contribute to the reduced eosinophilic inflammation. Importantly, the reduction of type 2–expressing ILC2s and T_H_2 cells in Gata-3^+/nlslacZ^ mice had no effect on the development of AHR in response to DEP+HDM. Interestingly, the modest inflammation that was observed in response to only HDM was also greatly reduced in the Gata-3^+/nlslacZ^ mice. In line, exposure of Gata-3 mutant mice to ovalbumin inhibited allergic airway inflammation.^45^ Moreover, therapeutic targeting of Gata-3 in a clinical trial involving allergic asthmatic patients also attenuated both late and early allergen-induced asthmatic responses.^46^

To further assess the relative contribution of the innate (ie, ILC2s) and adaptive (ie, T_H_2 cells) arm in our model of DEP-enhanced allergic airway inflammation, we used RORα^fl/fl^IL7R^Cre^ (ILC2-deficient) and Rag2^−/−^ mice, respectively. Intriguingly, we found that the DEP-enhanced allergic airway inflammation only tended to decrease in RORα^fl/fl^IL7R^Cre^ mice, whereas typical type 2 immune responses, such as eosinophilia, mucus metaplasia, and type 2 cytokine production, were completely abolished in Rag2^−/−^ mice that received combined DEP+HDM. In addition, Rag2^−/−^ mice exposed to DEP+HDM did not have AHR. This suggests that the presence of an adaptive immune system or at least an adequate interaction of adaptive immune cells with ILC2s is required in mediating the adjuvant capacity of DEPs on HDM-enhanced allergic airway inflammation and AHR. Of note, depletion of CD4^+^ or CD8^+^ T cells in a murine model with intraperitoneal exposure to DEPs plus ovalbumin was associated with abrogated cytokine production and ovalbumin-specific immunoglobulin responses in the peritoneal exudate fluid.^47^

Interestingly, although it was previously shown that ILC2s were critical in T cell–independent allergic lung inflammation,^17^ the modest inflammation in response to HDM in our model was completely abolished in the Rag2^−/−^ mice. Therefore the amount of administered HDM could be important, suggesting that doses of HDM that elicit limited biological inflammation on their own are unable to activate ILC2s and to drive the allergic airway inflammation in absence of an adaptive immune system. Furthermore, it could be that ILC2s are of less importance in subchronic responses than during the acute phases of an inflammatory response. This was supported by research performed in a papain model, in which the secondary responses were more likely T_H_2 dependent.^14^ Moreover, it was suggested that the communication between ILC2s and T_H_2 cells by a MHCII-mediated dialog or specific cytokine secretion could be crucial to substantiate their effects on allergic airway inflammation.^21^, ^22^, ^23^ However, it seemed that on concomitant DEP+HDM exposure, activation of T_H_2 cells appeared relatively independent of ILC2s, as opposed to previous reports in which ILC2s were crucial for the initiation of T_H_2 responses toward (relatively high) doses of HDM and papain.^14^, ^20^

Taken together, although a significant role for ILC2s has been demonstrated in several models of allergen-induced inflammation, our findings suggest that coexposure to multiple environmental factors, such as particulate pollutants and allergens (ie, HDM), modulate the contribution of ILC2s and T_H_2 cells to allergic airway inflammation and AHR.

**Key messages**

• Dysregulation of ILC2s and T_H_2 cells by targeting Gata-3 is associated with an attenuation of diesel-induced allergic airway inflammation.
• The adaptive immune system has a crucial role in diesel-enhanced allergic airway inflammation.
